# The Time Varying Networks of the Interoceptive Attention and Rest

**DOI:** 10.1523/ENEURO.0341-20.2021

**Published:** 2021-05-28

**Authors:** Ana Y. Martínez, Athena Demertzi, Clemens C. C. Bauer, Zeus Gracia-Tabuenca, Sarael Alcauter, Fernando A. Barrios

**Affiliations:** 1 Instituto de Neurobiología, Universidad Nacional Autónoma de México, Querétaro, QRO 76230 México; 2 GIGA Research Institute, University of Liège, 4000 Sart Tilman, Belgium; 3 McGovern Institute for Brain Research, Massachusetts Institute of Technology, Cambridge 02139, MA

**Keywords:** automatic classification, brain networks, correlation matrix, k means, attention, interoception

## Abstract

Focused attention to spontaneous sensations is a dynamic process that demands interoceptive abilities. Failure to control it has been linked to neuropsychiatric disorders like illness-anxiety disorder. Regulatory strategies, such as focused attention meditation (FAM), may enhance the ability to control focused attention particularly to body sensations, which can be reflected on functional neuroanatomy. The functional connectivity (FC) related to focused attention has been described, however, the dynamic brain organization associated to this process and the differences to the resting state remains to be studied. To quantify the cerebral dynamic counterpart of focused attention to interoception, we examined fifteen experienced meditators while performing a 20-min attentional task to spontaneous sensations. Subjects underwent three scanning sessions obtaining a resting-state scan before and after the task. Sliding window dynamic FC (DFC) and k-means clustering identified five recurrent FC patterns along the dorsal attention network (DAN), default mode network (DMN), and frontoparietal network (FPN). Subjects remained longer in a low connectivity brain pattern during the resting conditions. By contrast, subjects spent a higher proportion of time in complex patterns during the task than rest. Moreover, a carry-over effect in FC was observed following the interoceptive task performance, suggestive of an active role in the learning process linked to cognitive training. Our results suggest that focused attention to interoceptive processes, demands a dynamic brain organization with specific features that distinguishes it from the resting condition. This approach may provide new insights characterizing the neural basis of the focused attention, an essential component for human adaptability.

## Significance Statement

The dynamic brain connectivity related to focused attention to interoceptive processes remains to be explored. Here, we estimated the dynamic connectivity within the dorsal attention network (DAN), default mode network (DMN), and frontoparietal network (FPN) to characterize the focused attention to interoception and its differences to resting state. Five recurrent FC patterns were found. At rest subjects remain longer in a low connectivity pattern. In contrast to rest, the task showed an increase in the time spent in complex connectivity patterns. In addition, a lasting effect in the dynamic functional connectivity (DFC) that extended to the rest was observed following the task performance. Altogether, these results contribute to identify the dynamic brain organization supporting the focused attention.

## Introduction

In our everyday life, numerous stimuli surround us, requiring the selection, processing, and monitoring through focused attention (Posner, 2012; Farb et al., 2013). Once the stimulus is attended, it will be more likely to influence brain systems and to guide our behavior (Dehaene and Changeux, 2005; Webb and Graziano, 2015). Attention modulates the body representation in the somantosensory cortex. It facilitates the conscious perception of the external stimuli and also can increase or decrease the perception of spontaneous sensations, occurring without any external stimulus, as a response of the focused attention on the body (Boly et al., 2007; Ferentzi et al., 2018). Spontaneous sensations are related to interoception, the sensing of the body’s physiological condition, essential for body awareness (Michael and Naveteur, 2011; Michael et al., 2015). As a property of multiple cognitive processes (Bartolomeo and Chokron, 2002; Chun et al., 2011), the lack of regulation in the control of focused attention has an adverse impact on the metal health. It has been linked to psychiatric disorders (White and Shah, 2006; Donald et al., 2014), such as panic disorder, somatization, and illness anxiety disorders, distinguished by an excessive attention and increased concern in body sensations (Stins et al., 2015; Stern et al., 2017).

The ability to control focused attention particularly to body sensations may improve through regulatory strategies, like focused attention meditation (FAM) practices, considered a form of cognitive training (Tang et al., 2015). FAM requires to focus the attention to an object and bring the attention back to the object when it is lost (Manna et al., 2010), resulting in a better identification of the attention/inattention states (Lutz et al., 2008; Fox et al., 2016). Vipassana meditation is a practice characterized by focusing the attention on present-moment sensory awareness improving the attention control. In addition, Vipassana meditation laid the basis for the development of mindfulness techniques which have been used in clinical settings (Cahn and Polich, 2009; Goyal et al., 2014). Evidence suggests that such control of attention leads to changes in the functional neuroanatomical organization measured by functional connectivity (FC; Kilpatrick et al., 2011; Rabipour and Raz, 2012).

FC is an estimation of the communication across distant brain regions, which results in the integration of information (Friston et al., 1993; van den Heuvel and Hulshoff Pol, 2010), a fundamental principle for cognitive processes. Various FC systems have been linked to focused attention tasks, including the focusing to spontaneous sensations (Bauer et al., 2014), primarily the frontoparietal network (FPN; Bauer et al., 2019), the dorsal attention network (DAN; Corbetta and Shulman, 2002; Raz, 2004; Vossel et al., 2014), and the default mode network (DMN; Hasenkamp and Barsalou, 2012). The evidence of these studies suggests differences in the activation of these network regions related to the focus of attention, showing an increase activation of the FPN/DAN regions related to sustained attention and an activation of the DMN regions related to mind wandering (Hasenkamp and Barsalou, 2012; Hasenkamp et al., 2012). However, most of these fMRI studies have shown results which implicitly considered FC as stationary, i.e., representing an average of the FC from the scanning session (Hutchison et al., 2013a; Preti et al., 2017).

Recent FC studies have shown the dynamic nature of the brain activity, demonstrating connectivity patterns that evolve in time and organize in a hierarchical way (Vidaurre et al., 2017; Demertzi et al., 2019). However, the dynamic FC (DFC) analysis has focused, for the most part, on the investigation at rest. Therefore, although FC related to focused attention has been described, the dynamic modulation associated to focused attention and its differences with the resting state remains scarce (Fell et al., 2010; Hairston et al., 2017). The limited existing evidence about task DFC suggests a brain reorganization during tasks, with differences between the DFC patterns identified at rest versus motor tasks (Cheng et al., 2018), as well as differences in the frequency of the identified patterns during rest and during visually sustained attention tasks (Denkova et al., 2019), suggesting a response in the brain adaptation to cognitive demands (Gonzalez-Castillo and Bandettini, 2018).

Given these precedents, in this study, we aimed at quantifying the dynamic variations of FC between DMN, DAN, and FPN during three contiguous conditions; a resting state fMRI (rs-fMRI) session before a task, an spontaneous sensations attention task and during a rs-fMRI session after the task in experienced meditators. The inclusion of meditators provided the opportunity to explore the coordination of these networks during the interoceptive body focus in subjects with and advanced training in the control of this process.

The approach of this study can help to understand the dynamic brain organization underlying the focused attention to interoceptive processes, as well as the reorganization of the networks when going from a focused attention state to rest. This characterization offers an opportunity to obtain essential insights about the brain adaptation supporting this vital aspect of human adaptability and the brain reorganization according to the cognitive demands.

Based on the previous evidence, we hypothesized that this approach would allow to obtain the common DFC patterns that characterizes the dynamics of these networks during the three conditions. In addition, differences will be found in the frequency or time spent in these patterns as an effect of the ongoing experimental condition, with a higher time spent in patterns related to attention networks as DAN/FPN during the task.

## Materials and Methods

### Subjects

We included fifteen meditation practitioners in Vipassana meditation with an average number of hours of meditation practice at 1677 ± 367 h, six females, with a mean age of 40 ± 12 years old. All participants were evaluated for exclusion criteria: fMRI contraindications, history of psychiatric or neurologic disorder, or medical illness. Subjects answered digital versions of the Symptom Checklist 90 and Edinburgh Inventory to exclude psychiatric symptoms and to evaluate handedness, respectively. All participants signed an informed consent from the experiment approved by the Institutional Bioethics Committee, in accordance with the Declaration of Helsinki. At the end of the fMRI session, we applied an interview to assess the qualitative task experience of the subjects, to obtain information that ensured the subjects were awake and had an accurate performance of the instructions of the task.

### Experimental design

**Figure 1.**
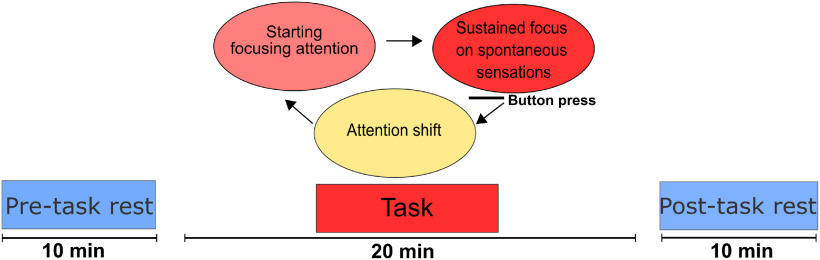
Experimental design. The fMRI data were acquired in three scanning sessions, starting with the 10-min pre-task rs scan, followed by 20-min task scan and the 10-min post-task rs. The task consisted in focusing attention to spontaneous sensations starting in the nostrils. Once a clear spontaneous sensation was felt, subjects sustained the focus on it for 3–5 s. Then pressed a button to signal the shift of attention to the next target.

Functional images were acquired in 1 d for each of the 15 subjects. Subjects underwent three functional scanning acquisitions starting with 10 min of rs-fMRI before the task (pre-task rest) scan, followed by 20 min of the focused attention task fMRI scan and finally 10 min of rs-fMRI after the task (post-task rest; Fig. 1). This implies three conditions for the subjects, however, one of the subjects did not conclude the post-task rs scan, therefore we obtained 14 subjects post-task rest data. The data for the pre-task rest and task condition was complete for the 15 subjects.

The task is a focus attention meditation technique, based in a previous study (Hasenkamp et al., 2012), which consisted of focusing the attention to spontaneous sensations (e.g., numbness, pulsation, tingling, warming, cooling, itching, tickle, vibration, flutter, skin stretch, stiffness, etc.) in five specific anatomic regions: nostrils, right thumb, left thumb, right great toe, left great toe, always in the same order, cyclically, and counterbalanced. Once participants started to feel a spontaneous sensation (i.e., pulsation) in the respective region, they were asked to sharpen focus more and more until they had a clear, distinct and uninterrupted sensation. When this level of felt sensation was reached, they were instructed to sustain it for ∼3–5 s and then press a button (Nordic Neurolab MR compatible button system). This button press signaled both the end of clear and distinct focus of felt sensation and shift of attention to the next anatomic region (Fig. 1). This was repeated at the participants own pace throughout the scan until the end. During the MRI session the subjects laid supine and remained relaxed with their eyes closed and avoided any motion. The time and the number of the responses were registered.

### MRI acquisition

Images were obtained using a 3.0T GE Discovery MR750 scanner (General Electric) with a 32-channel head coil. We acquired three fMRI scans during one session per subject: a resting state scan before the task (pre-task rs), then, the task scan and finally a rest scan after the task (post-task rest). The attention task scan was obtained using a T2* EPI pulse sequence of 20 min, with TR/TE = 1500/27 ms, 64 × 64 matrix, spatial resolution 4 × 4 × 4 mm^3^, 35 slices/volume, obtaining 804 volumes. The rs-fMRI scans consisted in an EPI pulse sequence of 10 min in duration each one, with TR/TE = 2000/40 ms, obtaining 300 volumes in the pre-task rest and 300 volumes in the post-task rest. During rest, subjects were asked to remain awake with their eyes closed. The TR for the attention task was selected according to the parameters of a previous meditation study (Hasenkamp et al., 2012) to adhere to the experiential sampling during meditation which required a faster acquisition. The differences in the TR as well as the differences in the length of the fMRI data between conditions has been shown to not affect the FC. The evidence of studies demonstrates that the strength of FC is stable with 6 min of fMRI data and is minimally affected by differences in many acquisition parameters including TR (Wu et al., 2011), with reliable results when common preprocessing procedures are applied (Van Dijk et al., 2010) . After the fMRI acquisition a high-resolution 3D T1 SPGR structural sequence was acquired (voxel size of 1 × 1 × 1 mm^3^, 256 × 256 matrix, TR/TE = 8.156/3.18 ms).

### ROI selection

According to the literature, focused attention and FAM practices are associated with changes in FC in the DAN, DMN, and FPN (Hasenkamp and Barsalou, 2012; Mooneyham et al., 2017; Bauer et al., 2019). Based on this evidence, we investigated the FC of these three networks. For this, we decided to use an individual parcellation approach, therefore, we created an individual mask in each subject containing the ROIs of the three networks, which would be used to make the FC analysis.

In order to create these individual masks, the pre-task rest data of each subject and the FC Toolbox (CONN) were used (Whitfield-Gabrieli and Nieto-Castanon, 2012).The steps for the individual ROIs mask procedure were taken as follows. First, the pre-task rest data subjects were preprocessed with a default preprocessing of CONN, which included motion correction, slice timing correction, segmentation, corregistration to the MNI152 standard space, artifact detection, regression and spatial smoothing of 6 mm. After this preprocessing, we performed a first-level fMRI connectivity analysis, specifically a voxel-to-voxel connectivity analysis in CONN for each pre-task rest data subject. For this we used we used 4-mm spheres, this size according to the spatial resolution of our data (isotropic 4 mm voxel). The spheres were created with SPM in MATLAB, centered for the DMN, DAN, and executive control network (ECN) regions based on the coordinates of Raichle (Raichle, 2011). This resulted in a pFDR map for each network per subject. These maps were thresholded with a *p* < 0.05 value and binarized, then, to eliminate voxels out of our regions of interest that could survived to the threshold, we used FSLtools to multiply the thresholded and binarized maps with the ROIs of the DMN, FPN/CEN, and DAN of the network atlas implemented in CONN. This last step allowed to obtain only the surviving voxels specific for the maps of each subject within our regions of interest and that overlapped with the area of the CONN ROIs. This CONN network atlas contains ROIs defined from CONNs ICA analysis in 497 subjects of the Human Connectome Project dataset (Whitfield-Gabrieli and Nieto-Castanon, 2012).

After this analysis we observed that the number of ROIs for the DAN mask differ between the subjects, given that for some subjects there were no surviving voxels in one of the frontal eye fields. However, the left and right intraparietal sulcus were present for the 15 subjects. Therefore, to homogenize the number of ROIs for the DAN mask between the subjects, we resolved to include only the left and right intraparietal sulcus as the defining regions of this network, given that the voxels in these two ROIs where above the threshold and present in the 15 subjects. The number of ROIs for the DMN and FPN did not differ between the subjects, with the FPN including four regions and the DMN four regions, for the 15 subjects.

**Figure 2.**
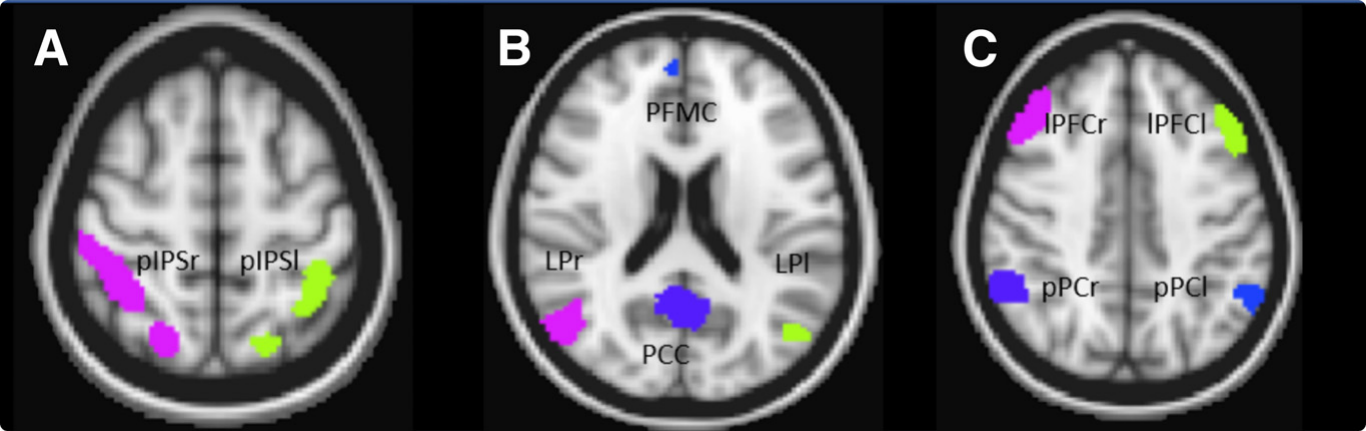
ROI masks obtained from a subject. The ROI masks of this subject included the DAN (***A***), DMN (***B***), and FPN (***C***) regions. These were combined into a single 10 ROIs mask which was used for the DFC analysis for this single subject.

The obtained three networks masks, per subject (Fig. 2), were combined into one mask with 10 ROIs, subject specific. These consisted in the left and right intraparietal sulcus, the medial prefrontal cortex (MPFC), posterior cingulate cortex (PCC), left and right lateral parietal cortex (LPl, LPr), the left and right lateral prefrontal cortex (LPFCl and LPFCr), and the left and right posterior parietal cortex (pPCl, pPCr; Fig. 2).

In regard to this individual masks procedure, we realize that there is no gold standard for regional parcellation (Fornito et al., 2010), with a variety of methods such as individual parcellations initiated from a group level scheme (Sohn et al., 2015) or group ROIs definition with the leave one-subject technique (Esterman et al., 2010) that could represent reasonable alternatives. However, we consider that this procedure brings the advantage of the ROIs identification directly from the individual data and according to their individual functional variations which may lead to a more accurate analysis of the FC and therefore a better identification of the DFC state (DFCS).

### Preprocessing

After obtaining the individual ROI masks for every subject, the pre-task rs, task fMRI, and post-task rs fMRI raw data, were preprocessed using FSL (Smith et al., 2004) and for this the structural images were required. The steps for this preprocessing were: extraction and discarding of skull and other non-brain tissue from the structural and functional image using BET of FSL (Smith, 2002) and reorientation. Slice timing correction, motion correction using MCFLIRT tool (Jenkinson et al., 2002), linear corregistration with FLIRT tool and nonlinear with FNIRT to the MNI152 standard space, segmentation of white matter and cerebrospinal fluid, regression of the signal from CSF, white matter and motion, artifact extraction with aCompCor (Behzadi et al., 2007) and bandpass filtering of 0.01–0.08 Hz. We did not perform the global signal regression since previous studies suggest that this may lead to false anticorrelations (Chai et al., 2012).

### DFC analysis

Sliding windows is a strategy applied to explore the time dynamic nature of the FC and in conjunction with a clustering approach as k-means, allows to identify patterns of FC that may reoccur in time across subjects, defined as DFCSs (Chang and Glover, 2010; Damaraju et al., 2014).

**Figure 3.**
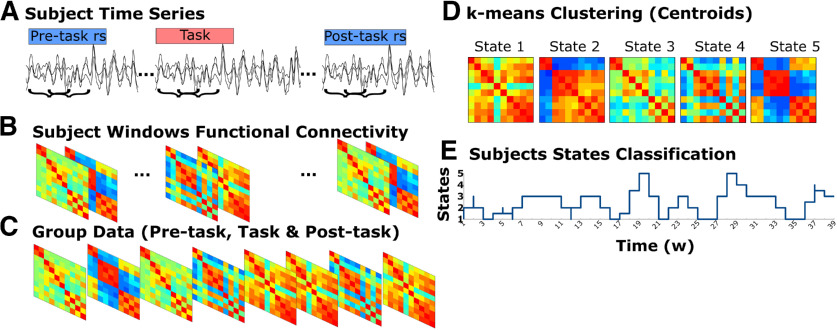
DFC analysis. ***A***, The time courses of the three conditions in each subject where used for the sliding windows analysis. ***B***, In each 30-s window the FC of the 10 ROIS was calculated. ***C***, The data from all subjects and conditions were joined into a group dataset. ***D***, The cluster solution of 5 was applied and k-means clustering was used to obtain the centroids of each cluster, which represented the DFCS. ***E***, Each window, which represented 30 s of time, was classified to one of the states, allowing to estimate the transitions and the time spent in the states for each subject and condition.

Applying a similar approach of previous studies (Damaraju et al., 2014; Mooneyham et al., 2017), we used sliding windows to determine the time varying FC (Sakoğlu et al., 2010) between the 10 ROIs of DAN, DMN, and FPN in the preprocessed data (pre-task rs, task, and post-task rs). Then, to estimate the DFCSs, we used the k-means algorithm for clustering (Chang and Glover, 2010; Allen et al., 2014; Fig. 3). For this DFC analysis, we used FSL tools, R software (3.4 version; R Foundation for Statistical Computing, 2016) and different R packages.

### Sliding windows and clustering approach

#### Sliding windows

For the sliding windows approach we established 30-s windows width and 15-s steps using fslsplit and fslmerge tools in the preprocessed data. The selected window size is according to previous studies describing a minimum windows size of 30 s recommended to capture the FC fluctuations (Hutchison et al., 2013b; Tagliazucchi and Laufs, 2015). Therefore, in the task data we obtained 79 windows of 30 s (20 TR) width and 15-s (10 TR) steps per subject, representing the 20 min of the subject task scan. This results in 1185 windows for the task group data. For the rest data, using 30-s (15 TR) windows width and 15-s (7 TR) steps, we obtained 39 windows for the 10 min of the pre-task rest and 39 windows for the 10 min of the post-task rest per subject. Therefore, in the pre-task rest group data we obtained 585 windows of the 15 subjects and in the post-task group data 546 windows of 14 subjects were obtained, since one subject did not conclude the post-task rest scan. The group windowed data, including the pre-task, task and post-task, consisted in 2316 windows. In each window we calculated the FC between the ten regions of the three networks using the ROI mask estimated for each subject. For the FC estimation we computed the Pearson’s *R* for each pair of ROIs, obtaining a 10 × 10 matrix of connectivity for each window, then these correlation values were transformed to z values.

#### Clustering

The 2316 FC matrices containing z values were grouped into a dataset that would be used to apply the k-means clustering. To determine the ideal number of clusters for the grouped dataset, we made an independent analysis. This consisted in determine the best number of clusters individually to the rest condition (pre and post-task rest) and to the task condition FC matrices using the NbClust package (Charrad et al., 2014) from R software. NbClust is a package that determines the optimal number of clusters in a dataset using 30 indexes. This determination is based on the majority rule in this package, which selects the optimal number of clusters based on the number of clusters proposed by most of the indices in the partitioning. We acknowledge that the selection of the optimal number of clusters is a difficult problem in clustering analysis, however, we considered that its definition using the majority rule was a reasonable solution for our study. The resulting best number of clusters for the rest data were three clusters according to 14 indices and for the task data were five clusters according to 10 indices. Then we applied k-means clustering independently to rest and task data with the three clusters solution for rest and five clusters for the task. We observed that the three centroids obtained for the resting condition shared similar values with three of the five centroids obtained for the task data. Therefore, we decided to use the five-cluster solution as the optimal number of clusters for the grouped dataset (pre-task rest, task, and post-task rest), allowing to fairly compare the dynamics between conditions and perform statistical testing on their differences.

After this estimation, we applied the k-means clustering in the grouped data, using the k = 5 solution, Euclidean distance, and 25 iterations for the algorithm, we obtained the five centroids of the five clusters; these centroids represented the five DFCSs. The clustering analysis also allowed us to classify each FC matrix from the dataset to one of these five states. Given that each matrix represents 30 s of time, we estimated the proportion of time spent on each state for each condition per subject. For this, in each subject we determined the number of matrices classified to a DFCS during a condition, this number was divided by the total number of matrices of the condition; this resulted in a time proportion for each state and for condition per subject, which then allowed us to estimate differences in the proportion of time spent on a state between the conditions.

In order to define whether the experimental condition had an effect over the proportion of time spent in the FC states, we applied the mixed effects linear model using the lme function from the nlme package in R (Pinheiro et al., 2017). This test was preferred since one of the subjects did not complete the 10 min of the post-task rs scan, therefore, we had a non-balanced sample. In the model fitting we used the proportion of time spent in each state by condition as the dependent variable and the condition and state interaction as the independent variable, with the subject by condition as the random effect. Then we tested the significance of the model effects with ANOVA. The *post hoc* comparisons were performed using the emmeans package from R software (Lenth et al., 2020) which obtains the estimated marginal means for the model and allows to compute the contrasts. The *p* values of the contrasts were then corrected for multiple group comparisons using the Bonferroni test with a significant level *p* < 0.05.

## Results

### Behavioral data

The button presses during the task which signaled the attention shift were registered for each subject. This resulted in an average number of responses of 20.5 ± 8.8. The 15 subjects indicated that they remained awake and performed the task as was instructed according to the information given by the subjects. In addition, one of the researchers supervised the absence of movement in the subjects during the performance of the task.

### DFCSs

**Figure 4.**
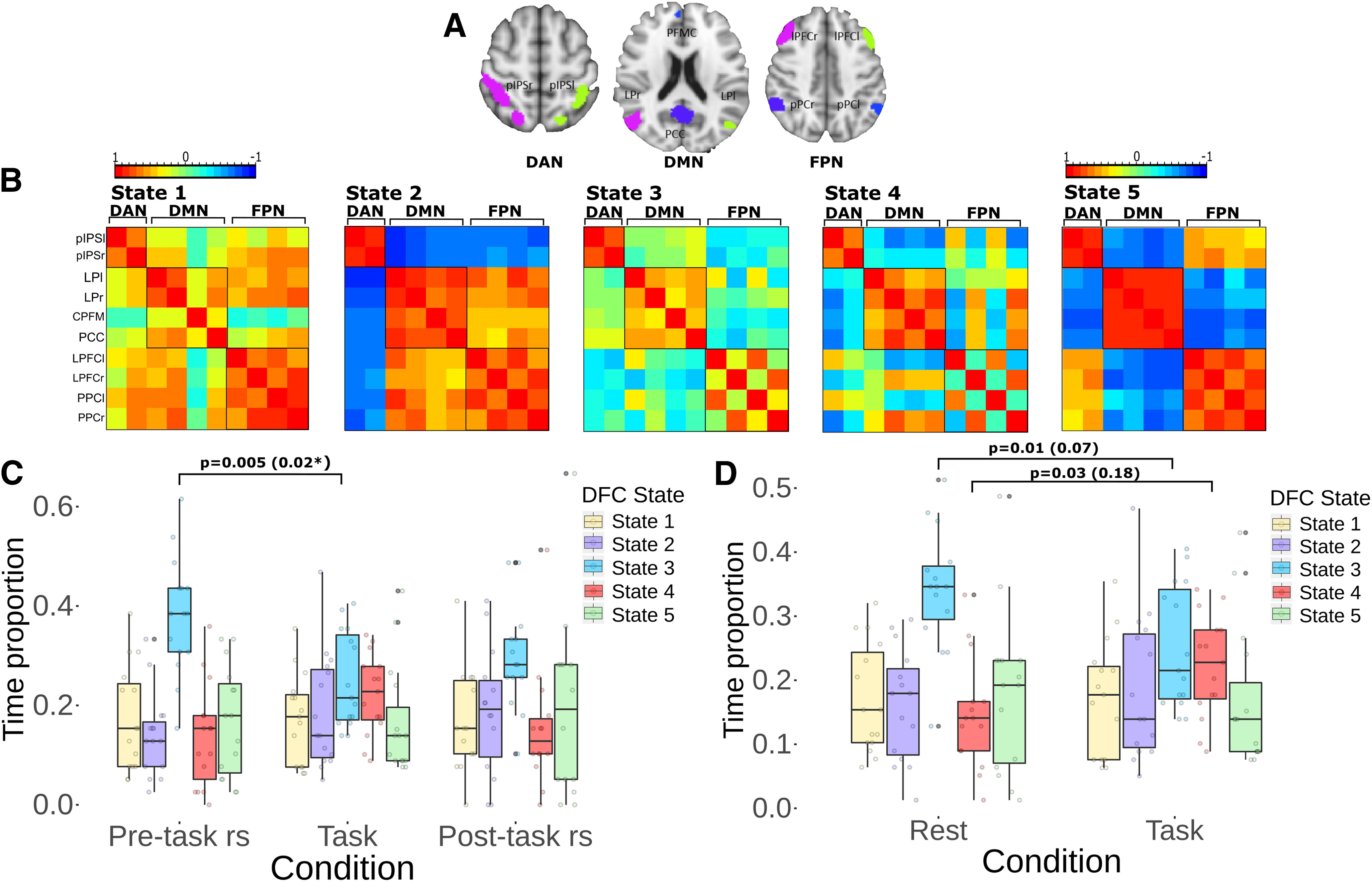
DFCSs ***A***, The individual masks where used for the analysis. ***B***, Five DFCS were obtained each one with a characteristic FC pattern and presented across the three conditions. ***C***, Boxplots of the proportion of time spent in each state shows differences between the three conditions. After being corrected, a significant decrease (*) in the time spent in the state 3 during the task performance in contrast with the pre-task rest was revealed. ***D***, An analysis contrasting between both rest conditions and task showed similar results, with a decrease in the time spent in the state 3 and an increase in the time spent in the state 4 during the task, after the correction did not show significancy.

Five DFCSs were identified, which are described here as states 1–5 and represent the pre-task rest, task and the post-task rest conditions (Fig. 4*B*). Each state characterizes a distinct FC between the 10 ROIs of three networks, DAN, FPN, and DMN, implicated in focused attention.

State 1 is characterized by a high connectivity between the regions of the three networks, except for the MPFC in the DMN, which shows low correlation values, near to 0, with the DAN and FPN regions. This state is also characterized by a preserved intranetwork connectivity. The state 2 shows a high connectivity between the DMN and FPN regions whereas, the regions from these two networks are anticorrelated with the DAN regions. Although the DAN is segregated from the two other networks, this shows a high intranetwork connectivity in this state. The state 3 demonstrates the lowest FC between the regions of the three networks in comparison with the other states. While this distinctive low internetwork connectivity points out a remarkable difference with the other states, it is also characterized by a preservation of the intranetwork FC. The state 4 is marked by the right FPN regions showing positive correlation values with the DMN regions, whereas the left FPN regions show positive correlation with the DAN regions. This distinctive finding was associated to a decoupling between the left and right regions in the FPN, showing extremely low connectivity values between them. The state 5 shows a high connectivity between the DAN and the FPN regions, whereas these two networks show anticorrelations with the DMN regions. Although this state shows a segregated DMN, this network exhibits the highest intranetwork connectivity in comparison with the other states.

The individual parcellations used for this study aimed to obtain the functional network regions specific for each subject that allows a more accurate analysis of FC and a better identification of the DFCS. However, given that the use of a common anatomic atlas to study group FC is an approach widely used in fMRI (Salehi et al., 2018), we decided to compare and quantify the degree of convergence between the resulting DFCS using ROIs of a standardized group atlas as the CONN network atlas with the DFCS using the individual parcellations. The CONN network atlas contains 32 ROIS defined from CONNs ICA analysis in 497 subjects of the Human Connectome Project dataset and has been used for previous FC studies (Whitfield-Gabrieli and Nieto-Castanon, 2012). We selected 10 ROIs from the DAN, FPN, and DMN of this atlas. These 10 ROIs which defined our group atlas consisted in the MPFC, PCC, LPl and LPr for the DMN, LPFCl and LPFCr, left and right posterior parietal cortex for the FPN, and the left and right intraparietal sulcus for the DAN.

**Figure 5.**
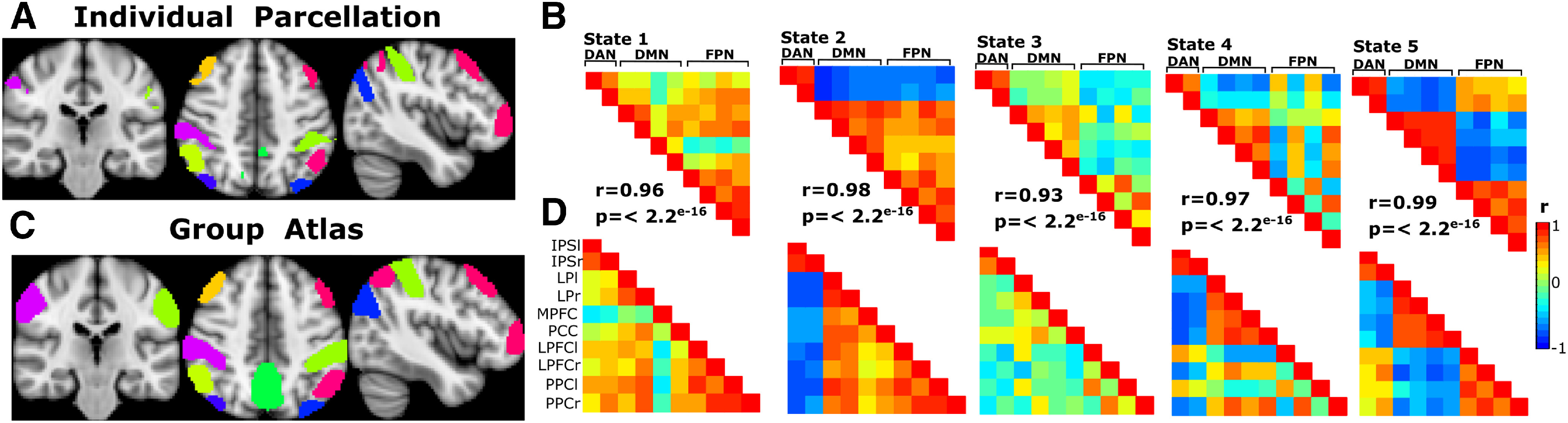
Strong correlation between the DFCS using individual parcellations and using a common group atlas. ***A***, The 10 ROIs mask obtained from a subject is depicted. ***B***, These individual parcellations were used to obtain the five DFCSs. ***C***, To assess the differences with a group common atlas, the 10 ROIS including DAN, DMN, and FPN ROIS of the CONN network atlas was used. ***D***, Obtaining the five states of the common atlas. The correlation (*r*) and significancy between the visually matched states is shown.

Using this group atlas, we applied the clustering approach with the five-cluster solution. We obtained the five DFCS and visually compare these correlation matrices with the obtained using the individual masks and it was observed that each of these five states had a matched state with one of the DFCS of the individual mask (Fig. 5). A correlation Pearson’s test was performed between the z-values of the paired matching states, observing a significant high positive correlation between the five pairs of matching states. The results were for the state 1 *r* = 0.96, *p* ≤ 2.2^e-16^, 95% confidence interval (CI) [0.93, 0.97], state 2 *r* = 0.98, *p* ≤ 2.2^e-16^, 95% CI [0.97, 0.99], state 3 *r* = 0.93, *p* ≤ 2.2^e-16,^ 95% CI [0.88, 0.96], state 4 *r* = 0.97, *p* ≤ 2.2^e-16,^ 95% CI [0.95, 0.98], state 5 *r* = 0.99, *p* ≤ 2.2^e-16,^ 95% CI [0.98, 0.99]]. These results demonstrate a high degree of convergence between the FC states obtained using individual parcellations and using a group atlas. This suggests that the individual approach used in our study shows a high consistency with the use of alternative parcellation approaches. However, it should be emphasized that for the individual parcellations procedure, the 10 ROIs from the CONN network atlas were also used, during the last step of their elaboration. This could partly explain the high degree of convergence between the results using both parcellations.

### Condition differences in DFC

The application of the DFC analysis to the grouped data not only allowed us to characterize the states that describe the brain dynamics of the three conditions, but also enabled us to investigate significant differences between them. These differences were investigated in relation to the proportion of time spent in each of the five states for each condition.

The pre-task rs condition consisted in 585 FC matrices of the grouped data, which were classified each one to a DFCS. The analysis of the proportion of time spent in each state for this condition resulted in a mean proportion of 0.172 in the state 1, 0.140 in the state 2, 0.381 in the state 3, 0.138 in the state 4 and 0.167 in the state 5. The task condition consisted in 1185 FC matrices that were classified to one of the five states. The calculation of the mean proportion of time resulted in a mean proportion of 0.172 spent in the state 1, 0.188 in the state 2, 0.251 in the state 3, 0.221 in the state 4 and 0.165 in the state 5 during the task condition. For the post-task rs condition there were 546 FC matrices used to the analysis of the mean proportion of time spent on the states. This resulted in a mean proportion of 0.168 in the state 1, 0.188 in the state 2, 0.294 in the state 3, 0.153 in the state 4, and 0.194 in the state 5 for this condition (Fig. 4*C*).

These results demonstrate differences in the proportion of time spent in each of the DFCS in relation to the condition, however, the differences were remarkable between the task and the rest conditions in relation to the states 3 and 4. In the case of the state 3, there is an evident decrease in the time spent in this low FC state during the performance of the focused attention task in comparison to the pre-task and the post-task rs conditions. On the other hand, during the task performance, there is a pronounced increase in the time spent on the state 4 characterized by a divided coupling of the FPN with the DAN and DMN, in comparison with the time spent in this same state during the pre-task rs and post-task rs conditions. Differences between the rest conditions were less pronounced, however, there is a decrease in the time spent in the state 3 and an increase in the time spent in the state 5 during the post-task rs in comparison with the pre-task rs.

The statistical analysis using the linear mixed effect model and anova to the fitted model shows a significant effect of the condition and state interaction over the time proportion (*F*_(8,191)_ = 2.1473, *p* = 0.03^a^), indicating that as predicted in our hypothesis, the proportion of time spent in these states is influenced by the ongoing attention condition. The *post hoc* comparisons shows that there is a significant difference in the proportion of time spent in the state 3 between the pre-task rs and the task condition (*p* = 0.005^b^, 95% CI, *d* = 1.146). After the correction for multiple group comparisons with Bonferroni test, we obtained a *p* = 0.029^b^ for this contrast. This indicates that there is a significant decrease in the time spent on the state 3, the low connectivity state, during the performance of the task in comparison to the pre-task rs condition. There were no significant findings for the time spent in the other states in relation to the condition.

As is shown in Figure 4*C* differences are predominantly and significant between the task and the rest conditions, for this reason, we compared both rest conditions as a single rest condition with the task, to assess whether the differences were more pronounced. The results show for the state 1 a mean proportion of time of 0.170 during rest and 0.172 during the task condition, for the state 2 a mean of 0.160 in rest and 0.188 during the task, the state 3 shows a mean of 0.341 in rest and 0.251 in task, the state 4 shows a 0.146 in rest and 0.221 during the task and the state 5 0.182 during rest and 0.165 in task. The Figure 4*D* illustrate these differences, which reflects once again a remarkable difference for the state 3 and state 4 between task and the rest condition. During the performance of the task there is a decrease in the time spent on the state 3, while during this same condition an evident higher proportion of time spent in the state 4 is presented in comparison with the rest condition. The statistical analysis with the linear mixed effects model again demonstrates a significant effect for the state and condition interaction (*F*_(4,126)_ = 2.8486, *p* = 0.026^c^), indicating that the proportion of time spent in the states is affected by the experimental condition. The *post hoc* comparisons between that rest-task contrast of the time for the state 3 resulted in *p* = 0.014^d^, *d* = 0.907 and for the state 4 resulted in *p* = 0.037^e^, *d* = −0.767 using a 95% CI. However, after the Bonferroni correction for multiple groups comparisons we obtained a *p* = 0.07^d^ and *p* = 0.18^e^ for the state 3 and state 4, respectively. There were no significant findings for the time in the other states in relation to these two conditions.

Given that the button press signaled the attention shift, we analyzed the correlation between the number of responses and the time proportion spent in the five DFCSs during the task condition. However, we did not find a significant correlation (state 1 *r* = −0.05, *p* = 0.83, state 2 *r* = 0.09, *p* = 0.73, state 3 *r* = −0.27 *p* = 0.32, state 4 *r* = 0.24, *p* = 0.38, state 5 = 0.01, *p* = 0.96) (Table 1).

## Discussion

In this study, we estimated the DFCSs of the DMN, DAN, and FPN during three different contiguous conditions, a pre-task resting state, a focused attention to spontaneous sensations task and a post-task resting state. The objective was to determine the dynamics of these three networks during the focused attention to interoceptive processes and to compare it with the brain dynamics of the resting state. We also aim to explore the effects of this task performance over the subsequent rest FC.

Our main finding was that there are five different patterns of FC characterizing the conditions, each state with a specific connectivity between the three networks. A dynamic transition between the states was presented over the course of the three conditions, consistent with previous work stating that a varying brain FC configuration is essential for changes in behavior and cognitive demands (Tagliazucchi and Laufs, 2015). Moreover the proportion of time spent in each state was significatively related to the ongoing experimental condition confirming our hypothesis and in line with previous evidence that suggest that the time spent in these states is not random and that it might be associated with behavior (Vidaurre et al., 2017). These differences were more evident between the task and the resting conditions, with the task showing a more complex organization in agreement with studies suggesting that task FC represents a combination of spontaneous activity and the task-related responses (Fransson, 2006; Fox and Raichle, 2007).

During the pre-task rest condition, these five states were present, consistent with previous studies showing that brain activity during resting conditions is not abolished, instead, it was characterized by a varied set of FC patterns. However, the subjects spent a significant higher proportion of time in the state 3 in comparison with the task condition. This demonstrate that although a dynamic interaction between the networks was present, the tendency was to remain longer in a low internetwork connectivity brain pattern at rest. This is in line with previous evidence revealing a higher frequency of a pattern characterized by a lower connectivity between DMN, salience network (SN) and CEN during rest compared with a visually focused attention task (Denkova et al., 2019).

This low connectivity state was the more prevalent across the three conditions, however, during the task, there was a significant decrease in the time spent in this state. Considering these findings and the evidence that demonstrates that the time spent in these states is associated with the information processing (Vidaurre et al., 2017), we suggest that this low connectivity state is a basal and a transitioning state that allows the response to processes. This conclusion is also supported by a previous study that determined the DFCS of the SN, CEN, and DMN during a breathing attention task, finding a state characterized by low correlations between the regions of the three networks and a preservation of the intranetwork arguing that this could represent a state that enables the transition from a state to another (Mooneyham et al., 2017).

In addition, the task condition was associated to a higher proportion of time spent in the state 4, in comparison to both rest conditions, with a trend toward significance. This state showed the right FPN regions interacting with the DMN and the left FPN regions with the DAN and a decoupling of the left and right regions in the FPN. The flexible interaction of the FPN with DAN and DMN of this state is consistent with evidence showing that this sequential interaction is fundamental for the control and adaptation in task demands (Cole et al., 2013; Harding et al., 2015). Although the FPN has been often viewed as a unitary system, the decoupling between its left and right regions is consistent with recent works showing two subsystems as an internal organization for this the network, one connected with the DMN and the other with DAN, which contributes to the ability to deliberately guide actions based on goals (Dixon et al., 2018). Moreover, the characteristics of this state agrees with previous FAM studies demonstrating that activity in FPN regions effects on the connectivity of the DMN (Bauer et al., 2019). This led us to suggest that this is a fundamental pattern of connectivity for the focused attention performance, conducted in a group of meditators. Although not significant, the task performance was associated to an increase in the average time spent in the state 2, distinctive by a strong connectivity between DMN and FPN regions with segregation of the DAN. While the FPN and DMN are usually assumed to work in opposition (Hsu et al., 2014; Crockett et al., 2017), evidence suggest that their interaction may be involved in the regulation of introspective processes independent from sensory input and spontaneous thoughts (Christoff et al., 2009; Fox et al., 2015). The regulation of this introspective processes is a key characteristic of the FAM practices which would be consistent with the higher proportion found for this state during the sustained attention to spontaneous sensations. In addition to these task estimations, we evaluated the correlation between the number of responses of the subjects and the time spent in the states during the task. However, our results showed no significant correlation. The responses signaled the end of the focus and the shift of attention, performed at the own pace of the subject. While the number of responses were not considered as a measure of accuracy for our task, they did reflect the reorienting of attention. Therefore, a correlation between the DFC measures and the number of responses was expected. Previous work has shown a correlation between attention task performance and the DFC metrics (Madhyastha et al., 2015), in contrast, other authors have shown no significant correlation between the task outcomes and the DFC results (Denkova et al., 2019). We consider that the lack of correlation in our results could be associated to the small sample size. In addition, the time spent in the states was the only DFC metric evaluated in our study, with other aspects of the DFC such as transition variability or shifts in graph properties (Gonzalez-Castillo and Bandettini, 2018) and their relation with the task responses not explored in our work, which could be an interesting line for future research.

The post-task rest condition showed small differences with the pre-task rest in relation with the proportions of time spent in the states. These differences consisted in a decrease time in the low connectivity state 3 and an increase in the average time spent in the state 5 (Fig. 4). Although not significant, the differences of the dynamic connectivity between the rest conditions are relevant since both shared the same cognitive instructions during the scan, except for the previous task performance in the post-task rest condition. The state 5 is a pattern that showed a strong connectivity between DAN and FPN, networks that have been associated to focused attention states (Tang and Posner, 2009; Madhyastha et al., 2015; Mooneyham et al., 2017). Our results showed an increase in the time spent in this state in the case of the task against rest comparison. The state 3, which showed low interaction between the networks decrease during the task performance. Therefore, the findings during the post-task condition in contrast to the pre-task rest, suggest a task-mediation effect extending to the post-task connectivity. Previous studies have already shown that the rest FC succeeding a task, is affected by the prior cognitive state (Waites et al., 2005). However, these results showing that the prior cognition also modulates the succeeding rest DFC, suggests that the dynamics might be influenced by repetitive tasks or interventions such as cognitive training. The lasting effect that extends into the non-attentive period leads us to question whether the time these effect lasts after the task supports the learning process of cognitive training techniques.

Over the three conditions, the state 1 characterized by a high connectivity between all the regions and a decoupling of the MPFC from the DAN and from the FPN was present in a similar mean proportion. MPFC supports self-related processing, emotional adaptive responses (Jang et al., 2011; Euston et al., 2012), and its connectivity is associated with conscious awareness (Luo et al., 2017). The MPFC is a DMN region, a network that has been consistently linked to mind wandering (Kucyi, 2018). The study of the role of this specific region related to mind wandering have suggested that the MPFC is a crucial region for mind wandering (Bertossi et al., 2017). With regards to meditation, studies have found MPFC activity linked to mind wandering during focused attention to breath (Hasenkamp and Barsalou, 2012; Hasenkamp et al., 2012). This previous evidence and the comparable distribution of this state across the conditions lead us to consider that it could represent a mind wandering state, where subjects experience non-directed thoughts.

In light of the findings and the previous evidence, we consider that the states found in our study represents the dynamic functional organization related to the cyclical attention states presented across the rest and the task performance.

Beyond the description of the focused attention FC, our results could represent the dynamic integration of these networks in relation to interoception and body processing because of the task characteristics, with the permanence in each of the states as a fundamental way to efficiently fulfill the cognitive demands. The association of the interoception processing to a wide coordination between cognitive control networks showed in our results, suggest a top-down control from these networks probably to primary cortical regions linked to interoception, such as the insula and somatosensory cortex (Bauer et al., 2014; García-Cordero et al., 2017). This conclusion is supported by anatomic findings indicating inputs from parietal and occipital cortices to the insula which in turn integrates somatosensory information (Namkung et al., 2017). Considering that we were particularly interested in quantifying the dynamic variations of three cognitive control networks we did not include the insula in our analysis; However, we consider that for future directions it would be interesting to study the dynamic coordination between these networks and the insula associated with focused attention.

Our findings also have significance in the field of meditation practices since the task we used consisted in a meditation technique. Meditation practices in particular focused attention techniques have been related to a better identification and control of attention (Lutz et al., 2008; Tang et al., 2015). These benefits has attracted the interest in their study (Lutz et al., 2014) and in their application, including the use of meditation techniques in clinical settings (Black et al., 2015; Wielgosz et al., 2019). Our results suggest that this practice engages a dynamic set of configurations of cognitive control networks, with a higher prevalence of more complex patterns that enables the required focus for this practice as well as an extending effect to the postmeditation rest period. This could explain the persistent changes associated to the expertise in this practice as the control of attention, emotion regulation, and less mindlessness.

The motor response of the button press could raise concerns over the effects of it on the resulting FC. Therefore, given that the sensory motor network (SMN) is particularly active in motor task, we decided not to include this network, to avoid its confounding effects on our results, although it has been also associated to spontaneous sensations in the absence of motor processes. Limitations of the study included a small sample size; However, this encouraged to use an individual parcellation approach, additionally, using the sliding windows analysis we were able to obtain a large amount of data points for the FC in each subject, that was included for the statistical analysis. The lack of a control group difficult the generalization of the results, this motivated the design and the comparison to the resting condition. We realize the control group would enable the conclusions in a standard population, therefore, for future directions we propose to address these implications and additionally to include primary regions associated to interoception for characterize the brain coordination related to this interoceptive attention process. The characteristics of the sliding windows analysis were chosen for the comparison between the task and rest conditions, without considering the moment of the button press to define cognitive intervals for the task (Hasenkamp et al., 2012). Further analysis could take this implication into consideration. This could provide a better understanding of the relation between the task outcomes and the DFC measures during the task. Additionally, we did not explore the interindividual variability of the FC; however, we consider that the address of this issue in future research would offer further insights into the interindividual variability of the dynamic connectivity.

Altogether, the FC states found in our study and the time spent on them characterizes the brain connectivity modulation of the focused attention to interoception, as well as the FC modulation of the changing cognitive demands of going from a resting condition to a focused attention state. The impact of this task performance over the subsequent rest brain dynamics helps to understand that the dynamic brain organization might be influenced through external demands and that this effect extends for a period. This raises as a possible underlying mechanism of the functional changes associated to repetitive focused attention interventions. Future research should aim to study the modulation on the brain dynamics through these forms of attention interventions in health and for clinical settings.

**Table 1:** Statistical table

	Data structure	Type of test	Statistic	CI	Effect Size
I	Condition differences in DFCS				
a	Effects of condition × state interaction on the time spent in the states	ANOVA of LME	*F*_(8,191)_ = 2.1473, *p* = 0.03		
	*Post hoc* comparisons					
b	State 3 pre-task rs task	EMM	df = 191, *t* = 3.139 *p* = 0.005 (0.029)	0.95	*d* = 1.146
II	Condition differences in DFCS					
c	Effects of condition × state interaction on the time spent in the states	ANOVA of LME	*F*_(4,126)_ = 2.8486, *p* = 0.026		
	*Post hoc* comparisons				
d	State 3 rest task	EMM	df = 126, *t* = 2.484 *p* = 0.014 (0.071)	0.95	*d* = 0.907
e	State 4 rest task	EMM	df = 126, *t* = −2.103 *p* = 0.037 (0.187)	0.95	*d* = −0.767

Statistical analysis to test the differences in the DFCS between the pre-task, task and pos-task rs condition (I) and between the rest and task condition (II). Letters refer to the *p* values reported in the results. The Bonferroni adjustment for the comparisons is indicated next to the *p* values. LME linear mixed effects model, EMM estimated marginal means for linear mixed effects model.

## Synthesis

Reviewing Editor: Erica Glasper, University of Maryland Decisions are customarily a result of the Reviewing Editor and the peer reviewers coming together and discussing their recommendations until a consensus is reached. When revisions are invited, a fact-based synthesis statement explaining their decision and outlining what is needed to prepare a revision will be listed below. The following reviewer(s) agreed to reveal their identity: Satoru Hiwa.

Paper #eN-NWR-0341-20

Synthesized Comments to the Authors

Overview: This study examined the dynamics of interoceptive attention during a functional connectivity task that consisted of an attention task preceded and followed by a resting-state condition. Results demonstrated that a higher prevalence for the low connectivity state during rest and a more complex state during the attention task; however, additional results (i.e., behavioral data during the tasks) would be beneficial. Overall, the study has some potential to inform the field of dynamic functional connectivity and, importantly, the role that focused attention has on intrinsic functional connectivity following a task. The experimental design and findings are interesting but there are some concerns related to the low N size. Also, the authors should be able to clarify/explain and justify their analyses approach (i.e., ROI selection) as well as to check for confounding effects (i.e., different lengths of scans, motor response during task). Additionally, the authors should clarify their hypotheses and explain how this informs prior research (given that the authors have previously published studies comparing rest and attention task dynamic connectivity). Additionally, there is a lack of methodological details.

The synthesized comments from both reviewers are provided below:

Major Comments

Introduction

1. The introduction would benefit from streamlining along with a discussion of relevant prior research using a similar task (see Hasenkamp et al.,2012, Hasenkamp & Barsolou, 2012) as well a research comparing dynamic functional connectivity during rest and attention (Denkova et al., 2020; Cheng et al., 2018; Kucyi et al., 2020).

2. It is important for the reader to clearly point out how the research is innovative. If the work was intended to be a replication of prior findings, this should be explicitly stated. Additionally, the introduction would benefit from a clear statement of the hypotheses and expected results.

Materials and Methods

1. The sample size (N = 15) is low. Authors should provide sound justification for this small number of participants.

2. Concerns surrounding experimental design should be directly addressed:

a. Potential confounding effects of the button press during the focused attention task may exist. Authors should discuss this.

b. The task and resting conditions are different lengths. Specifically, the resting condition is shorter than the task itself. Authors should discuss whether some of the observed differences are due to the different lengths of the conditions.

3. Clarity surrounding the description of the ROI methods in needed:

a. Are ROIs based on Raichle coordinates or the Functional Connectivity Toolbox atlas?

b. In the ROI selection procedure, authors applied individual parcellation and this approach leaves the authors wanting further clarification.

c. Authors should also provide possible explanations for their ROI mask determination method. For example, it is unclear why the authors did not make a group ROI mask using a leave-one-subject-out approach (see Esterman M, Neuroimage. 2010;50(2):572-576) because of the final integration of subject-wise functional connectivity matrices into the group dataset.

d. The authors observed the different number of ROIs for the dorsal attention network (DAN) mask between the subjects, however, this resulted in defining it as two regions. This should be justified.

e. The authors have overstated that only L/R intraparietal sulcus are defined as DAN. Authors should clarify whether SPL, associated with top-down orientation, was observed.

f. Authors should include a description of the variability in the number of ROIs for the remaining two networks, DMN and executive control network.

4. Detailed explanation for the inter-individual variability of the three networks, along with the results, should be provided.

5. Authors should explain why 4mm spheres were used?

6. Authors should provide the Bonferroni-corrected threshold.

7. It is unclear why the other metrics resulting from the dynamic functional connectivity analyses were not provided.

Results

1. Authors should include a definition of the group atlas and include the selection bias because they have already observed the individual atlas (this is described in line 285-293; however, this should be moved to the ‘Materials and Methods’ section). Instead of the current choice, authors should define it by widely using regions in many of the existing studies.

2. Authors should justify why only ‘time in responses’ was only reported and other measures were not reported.

3. For greater transparency, behavioral data from the task should be included.

4. It is unclear why the authors chose different TRs each for the resting and task blocks. This choice may have affected the comparison between resting-state and task conditions. There may be some confusion as to what the TR parameter indicates. It does not mean just a sampling time of the brain activity in fMRI since different TRs gives different S/N ratio.

5. The authors should provide clarity to the clustering analysis:

a. Line 229-230, Page 11: Please define the “majority rule”.

b. Why did the authors choose five clusters referring to the task condition while ignoring three clusters observed in the rest data?

c. Which indices of the NbClust package was chosen for the number of clusters determination?

d. What is the definition of ’optimal’ in the number of clusters? There is likely no optimal way to determine the number of clusters in data clustering methods, therefore, the authors should explain their assumption based on their definition of ‘optimal” in this study.

e. Which distance metrics are used for the k-means clustering? How many runs are executed for the k-means analysis for each data set? This will strongly affect the clustering results as well as the characteristics of centroids derived.

6. Authors recorded the signal of the attention shift, however there was no mention of this potentially interesting behavioral metric. The authors are encouraged to examine the relationship between the time proportions of the five FC states and the attention shifts among the five anatomical target regions.

7. If collected, the authors are encouraged to report data regarding the performance of meditation?

a. How did the authors ensure that participants performed their meditation correctly (e.g., staying awake during meditation)?

b. Did the authors use questionnaires to assess participants’ characteristics and how well they meditated? If available, this could assist the authors in ensuring the reliability of their results.

8. Authors should discuss why the insula was not involved in their results.

Discussion

1. Greater discussion of the results in light of the existing literature should be included. For example, main contributions of this study to contemplative sciences need to be better clarified and summarized.

Minor Comments

1. Line 183--184, Page 9: Authors should include literature to support the following claim: “We did not perform the global signal regression since previous studies suggest that this may lead to false anticorrelations.”

2. Please use the same terminology for ‘resting state’ throughout the document (rs or rest?).

3. Line 212--214, Page 11: Please be consistent with the terms TR or TRs.
